# Perceptions of stakeholders about the role of health system in suicide prevention in Ghizer, Gilgit-Baltistan, Pakistan

**DOI:** 10.1186/s12889-020-09081-x

**Published:** 2020-06-23

**Authors:** Anila Anjum, Tazeen Saeed Ali, Nousheen Akber Pradhan, Murad Khan, Rozina Karmaliani

**Affiliations:** 1 Department of Community Health Sciences, The Aga Khan University, Karachi, Pakistan; 2 School of Nursing & Midwifery, The Aga Khan University, Karachi, Pakistan; 3 Department of Psychiatry at The Aga Khan University, Karachi, Pakistan; 4 School of Nursing & Midwifery & Community Health Sciences, The Aga Khan University, Karachi, Pakistan

**Keywords:** Perceptions, Role of stakeholders, Health system, Suicide prevention, Strategies, Challenges

## Abstract

**Background:**

Suicide is a serious global public health problem, with more than 800,000 people dying by suicide worldwide every year. 79% of suicides happen in Low and Middle-Income Countries (LMICs), where lack of national suicide prevention programs coupled with inadequate MH facilities for early identification and treatment of mental disorders add to seriousness of the problems. Although there is paucity of research, studies suggest that the rate of suicide in district Ghizer, Gilgit-Baltistan (GB), in northern Pakistan may be higher compared to rest of the country.

**Methods:**

This study aimed to explore the perceptions of stakeholders about the role of the health system at District Ghizer, GB using a qualitative descriptive exploratory research design. A total of 12 face to face in-depth interviews were conducted from the stakeholders using purposive sampling technique.

**Results:**

The study results led to three broad themes, 1) Suicide as A Social Issue, 2) Role of Health System in Suicide Prevention, and 3) Challenges for Health System in Suicide Prevention. Theme one was sub-categorized into; a) Perceived situations contributing to suicide, b) Environmental factors. Theme two was subdivided into; a) Major hurdles for Health system, b) Lack of MH services in the available health system. Theme three was subdivided into; a) Lack of collaboration across-sectors, b) Unavailability of MH professionals, and c) Financial issues. The study findings reveal that there are multiple challenges for health system including, lack of awareness on mental issues, shortage of resources and lack of collaboration in the community. Moreover, existing policies or strategies need to be modified to overcome the existing challenges for the effective prevention.

**Conclusion:**

This study emphasized creating awareness about MH issues, introduction of school health programs, parental counseling session and strengthening of the health system by allocating suitable budget for MH issues and suicide prevention strategies.

## Background

Suicidal behavior includes suicidal ideation, self-harm attempts and completed suicide. According to the World Health Organization (WHO), suicide is a serious global public health issue, with an estimated 800,000 deaths worldwide WHO also estimates that for every suicide there are at least 10–20 acts of self-harm and 100 people have suicidal ideation [1]. 79% of the global suicides take place in Asia with only two countries, China and India contributing approximately 40% of the global suicides [2]. Suicide is strongly condemned in Islam and this is reflected in relatively low rate in Islamic countries [3].

Pakistan is a South Asian low and middle income country (LMIC). Its current population is 207.8 million [4]. 97% of the people are Muslims, 65% live in rural areas and a third under the poverty line. The country has four provinces i.e. Punjab, Sindh, Baluchistan and Khyber Pakhtunkhwa (KP) as well as the newly created province of Gilgit-Baltistan (GB). Pakistan is ethnically very diverse, has a number of cultures and sub-cultures and several languages, though Urdu is the national language and is widely spoken and understood. MH (MH) services are poorly resourced and organized in Pakistan. The psychiatrist to patient ratio is 1 to 0.5–1 million people. This is despite the fact that a systematic review showed that prevalence rates of 34% for common mental disorders (CMDs) were as high as 34% [5]. The economic burden on mental illnesses in Pakistan is estimated to be US$ 4.2 billion annually [6].

Suicidal behaviors are understudied and under-researched subjects in Pakistan [7]. However, over the last few years a number of studies have been published on the epidemiology and risk factors of suicidal behaviors in Pakistan. A study using secondary data determined suicide rates of six cities of Pakistan and showed that highest the rate of suicide in Rawalpindi (2.86 per 100,000) in 2006 and lowest in Peshawar (0.43 per 100,000) in 2000. The study also showed rates in men as 7.06 per 100,0000 and women as 3.08 per 100,000 in the Sindh province [8]. A recent psychological autopsy study from KP province identified major depression, use of harmful substances, lack of treatment compliance, financial issues, aggression, low self-confidence, family disputes and thoughts of self-harm as risk factors for suicide [9].

Over the last few years there have been anecdotal reports of increasing incidents of suicides, particularly of female suicides, in Gilgit-Baltistan (GB) in the Northern areas of Pakistan [10]. A study on female suicides in Ghizer district, GB showed crude rates as 14.89 per 100,000 and an annual age-specific death rate for young females (15–24 years) as 61.07 per 100,000 for the years 2000 to 2004 [9]. An ethnographic study explored major factors associated with female suicides in GB and them as 1) Social factors including academic pressure, relationship problems, divorce and financial issues, 2) Cultural factors including lack of freedom, lack of decision making power and male child preference and 3) Psychological (mental illness and depression) [11]. Another qualitative study in district Hunza GB revealed educational failure, psychological disorders, domestic issues, the transformation of joint to nuclear families and poverty among youth as risk factor for attempting suicide [11, 12]. Common methods that have been used in suicides in Ghizer includes jumping in rivers (40%), ingestion of poison (33%) hanging from the ceiling (6%), gunshot (5%) and jumping from height (2%) [7, 13]. A study has revealed that all of females attending a health center, 50% had depression/ anxiety [14].

### Role of the health system and suicide prevention

A n effective and comprehensive suicide prevention strategy comprises of a systems approach that integrates community and healthcare services [15]. Such a strategy focuses on collaborations across different sectors and addresses all the factors, including people, procedures, policies and organizations in improving the health of the community [16]. There are many difficulties in establishing suicide prevention programs in LMICs. A qualitative study on suicide surveillance and health systems in Nepal, revealed the that suicide data was filtered through a variety of reporting systems shaped by social, cultural, legal and medical institutions and that a suicide prevention strategy will not be possible without reliable statistics and standardized reporting practices [17]. A study in Cameroon in Africa showed that although suicide was not a rare event in rural settings, it was under-recognized and that only 13% (2/15) of consulting nurses were aware of three symptoms of depression or that it can lead to suicide. Unavailability of medications and proper guidelines to manage mental illnesses in the healthcare facilities were other issues highlighted [18].

A community based suicide prevention program for adolescents was implemented in town high schools of Chile [19]. Four hundred nine individuals including students, teachers, family and health professionals were given training for identification suicide risks in adolescents. Results showed that 20% risk individuals at risk of suicide were identified through the screening system, who were not identified by health system [20]. While the above studies reveal deficiencies in suicide prevention strategies in LMICs, they also identify potential areas for establishing and/or strengthening suicide prevention activities using a systems approach.

### Health system of Pakistan

The health system of Pakistan is quite diversified and includes public, private, para-statal, philanthropic contributors, civil society, and donor agencies. A major strength of public health system is an outreach primary health care, delivered at the community level by the lady health worker (LHW), lady health visitor (LHV) and community midwives, who have earned success and trust in the societies. In addition, the private sector serves to 70% of the community through a diverse group of qualified health team members to traditional faith healers [4].

According to the WHO-AIMS report of 2009 [21], MH services in Pakistan consists of 3729 outpatient (OP) facilities, of which 1% are for children and adolescents. 46% of OP facilities provide community-based follow-ups. There are 624 community-based psychiatric inpatient units for a total of 1.926 beds per 100,000 population. There are 0.2 per 100,000 psychiatrists, 8.13 per 100,000 nurses, 0.28 per 100,000 psychologists and 0.013 per 100,000 occupational therapists [22] Only 0.4% of the healthcare budget is allocated to MH by the government [22].

The health system of Gilgit-Baltistan is poorly developed. There are critical challenges with scarcity of resources in the area. The ratio of doctor to population is 1:4100 whereas, national ratio is 1:1206. MH issues are neglected, partly due to lack of awareness but also due to lack of MH facilities. This in turn contributes to lack of suicide prevention program, especially for secondary prevention. There is only one psychiatrist for the entire region, as well as unavailability of other trained healthcare professionals in the region [23].

### Rationale of the study

Although the prevalence and causes of suicide has been well researched in a number of LMICs, there have been no studies on the role of health system in the prevention of suicide in Gilgit-Baltistan. This will be the first qualitative exploratory study that assesses the role of health system in the prevention of suicide and identify gaps in the system that need to be addressed for suicide prevention. It is hoped that study will lay the groundwork for future studies and that will provide evidence to inform policy, program managers, non-governmental organizations (NGOs) health ministries and the local community to prevent suicides in the region. The aim of this study was to explore the perceptions of stakeholders about the role of the health system at District Ghizer, Gilgit-Baltistan.

### Research question

What are the Perceptions of Stakeholders about the role of the Health System in Suicide Prevention in Ghizer?

## Methods

### Research design

A descriptive exploratory qualitative research design was used to answer the research question. The aim of selecting a descriptive exploratory design is that it will give more insight to understand the capacity and role of the health system in Ghizer district (refer to additional file B for more details).

After doing a comprehensive literature review, a semi-structured interview guide (refer to the additional file A) was formulated for In-depth interviews (one to one) of key informants. Data was collected using a semi-structured interview guide. The interview guide contained four broad categories having 18 open-ended questions and most of them had probes. However, the researcher also used some unplanned probes to clarify the questions and to explore perceptions of the stakeholders.

The interview guide was developed by the researcher in consultation with thesis committee members. Questions were developed after reviewing the literature and the content was reviewed by the thesis supervisor and the committee members. After Ethics Research Committee (ERC) approval, pilot testing was performed on 10% of the sample, before starting data collection.

### Study setting

The study was conducted in the Ghizer district of Gilgit-Baltistan, in the Northern area of Pakistan. The district shares a border with China in the north, Chitral district in the West and District Gilgit to the East. It consists of four tehsils (administrative areas): Puniyal, Ishkoman, Gupis, and Yasin. The total population is 156,000, comprising of 91% rural area and 9% urban. The literacy rate of Ghizer is 96.39% [16].

### Study population/participants

The study participants consisted of the key stakeholders comprising of teachers, health-care professionals (doctors, nurses, midwives), police officers, media personnel, District Health Officer (DHO), religious leaders (Masjid Imam-Sunni) and (Mukhi-Ismaili community), youth, local community members (parents) and NGOs. The reason for interviewing such a diverse group was that suicide is the multidimensional phenomena and its prevention demands a multi-sectoral involvement [1].

### Sample and sampling

Purposive sampling technique was chosen for stakeholder selection; one stakeholder from each sector was interviewed. Purposive sampling requires in-depth analysis and a smaller number of study participants [24]. A total of 12 key informant were interviewed. Descriptive qualitative studies have a smaller sample size as compared to other studies, the typical sample size ranging from 5 to 20 respondents.

### Eligibility criteria

The study participants were recruited based on the following inclusion and exclusion criteria.

Inclusion Criteria:

- Stakeholders who can play a role in suicide prevention
- Participants who were willing to participate in the study.
- Stakeholders residing in district Ghizer at least for 3 years

Exclusion criteria:

- Stakeholders newly transferred from other cities or provinces to Ghizer.

### Data collection methods

Face to face in-depth interviews were conducted with each participant to gather the information by using a self-developed semi-structured interview guide. The purpose of selecting Key Informant Interviews was to obtain crucial information about the role of the health system in suicide prevention from stakeholders of Ghizer.

Data collection was started after getting approval from the ERC, Aga Khan University (AKU), Deputy Commissioner (DC) and the Aga Khan Health Board (AKHB) of the district Ghizer. Participants were informed about the research study via cellphone and SMS prior to the interview. Participants were asked about feasibility of interview setting, date and preferred language. All participants preferred the Urdu language. Interviews lasted between 35 min to an hour. Digital audio recording was used for transcribing, coding, and analysis after obtaining informed written consent. At the end of the interview, information was summarized and feedback taken from the participants to clarify understanding of the information provided.

### Establishing rigor of the study

We followed Lincoln and Guba’s criteria (1985) to ensure trustworthiness and rigor in the study. This includes credibility, confirmability, dependability, and transferability.

### Ethical considerations

The research was approved by the ERC, AKU Karachi, Pakistan. Permission letter was also taken from the DC of Ghizer district. Informed consent was obtained from each participant after explaining the purpose of the research prior to the interviews. Purpose of the study, benefit, and risks, and their right to participate were shared with participants. Participants were informed their participation was voluntary and could be withdrawn at any stage. Anonymity and confidentiality of the respondents were maintained throughout the study by giving codes and identity of each participant kept anonymously. The audiotaped data will be saved for a few years after the completion of the study. To ensure the safety of the data, soft and hard data were placed in lock and password secured. The rough data was only accessed by the primary researcher, supervisor, and committee members.

### Data analysis plan

Qualitative analysis is an ongoing, iterative process of data ordering, structuring, and developing concepts or meaning. It starts from the initial stages of the data collection phase and continues throughout the study [17, 25]. Data analysis were done manually and simultaneously with data collection to validate the manually analyzed data [26]. The observations, feelings and original comments were restructured and audiotape interviews were transcribed. Coding was used to analyze the data, similar codes were assigned a category and subcategory, and then themes were developed from the categories. The interpretation of the transcript was done following the steps of content-analysis provided by Creswell [25], which includes data reflection, data organization, and data coding process, data representation, data categorization, and data interpretation. In this research study, data organization, data reflection, and data coding were used.

1. Data organization: In this step, data were organized in a separate folder, which contained the demographic profile of the study participants, field notes, and researchers’ reflections.
2. Data Reflection: In this step, the interview transcripts, field notes, reflections, and other supporting papers were review and reflected with the supervisor. Transcripts were verified by listening to the audiotaped interview of the participant.
3. Data coding process: In this step data analysis began with the coding process. Transcripts were reviewed and similar meaning was quantified same color and codes.

## Results

The result consisted of the analysis of data gathered from the 12 in-depth interviews of key informants at district Ghizer is divided into two parts, demographic characteristics of the study participants and the main findings of the study derived from the interviews.

### Demographic information of the participants

A total of 12 stakeholders were recruited for this study (Table 1). Majority of the participants were males, with age range of 25–55 years. Most participants had a Master’s degree. Duration of work experience of the participants were as follows: 10–15 years- 42%; 20–25 years 25%; 05–10 years- 17%; 15–20 years-8%, while 8.0% had no work experience.

**Table 1:** Demographic information of the participants

**Characteristics**	**No. of participants (*N*** **= 12)**	**Percentage (%)**
**Sex**		
Male	11	91.6
Female	01	8.3
**Age (In years)**		
25–35 years	4	33.3
35–45 years	3	25.0
45–55 years	4	33.3
55–65 years	1	8.3
**Level of Education**		
No experience	0	0.0
Primary	0	0.0
Secondary	1	9.0
Matriculation	1	8.0
Intermediate	1	8.0
Bachelors	3	25.0
Masters	6	50.0
**Years of Experience**		
Nil	1	8.0
05–10 years	2	17.0
10–15 years	5	42.0
15–20 years	1	8.0
20–25 years	3	25.0

### Findings of in-depth interviews

**Fig. 1:**
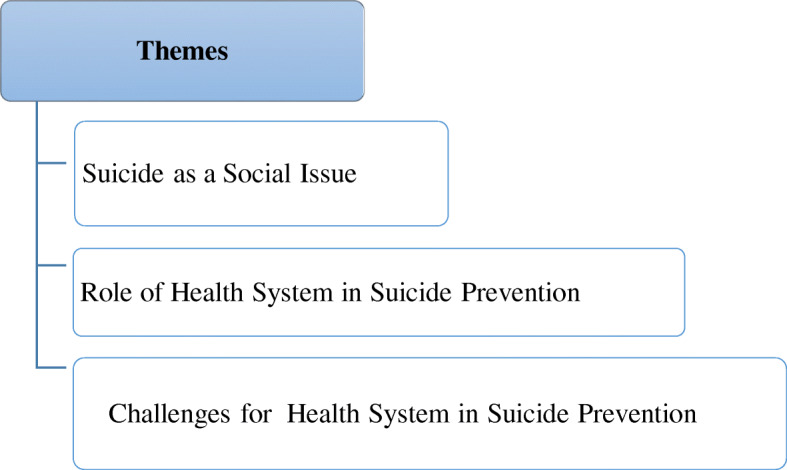
Major themes of the study findings

There are three major themes (Fig. 1) of the research study followed by categories and sub-categories (Table 2). The first theme is “Suicide as a Social Issue”, that illustrates the general view of stakeholders about suicide followed by perceived situations contributing to suicide incidents. This theme also consists of environmental factors that participants felt influence suicide.

**Table 2:** Themes, categories, and sub-categories of the study finding

***Themes***	***Categories***	***Sub-Categories***
**1. Suicide As a Social Issue**	1.1. Views of Stakeholders about Suicide	
	1.2. Perceived situations contributing to Suicide	1.2.1. Competitive environment and high expectations from adolescents
		1.2.2. Low resilience ability
		1.2.3. Domestic violence
		1.2.4. Family dynamics
	1.3. Environmental Factors	1.3.1. Clashes between traditional and modern environment
		1.3.2. Influences of Media on Society
**2. Role of Health system in Suicide Prevention**	2.3. Major hurdles for Health System	2.3.1. Lack of awareness about MH
		2.3.2. Unpredictable Suicide Behavior
	2.4. Lack of MH Services in available Health System	
**3. Challenges for Health System in Suicide Prevention**	3.1. Lack of Collaboration across-sectors	
	3.2. Un-availability of MH Professionals	
	3.3. Financial Issues	

The second theme is the “Role of Health system in Suicide Prevention”, which consists of the MH services and barriers to health system. The third theme, emphasizes on the “Challenges for Health system in Suicide Prevention”, where different challenges for the health system in Ghizer district will be discussed.

### Themes 1: Suicide as a social issue

This theme is divided into three categories, the general views of stakeholders about suicide; perceive situations contributing to suicide and environmental factors responsible for suicide cause in Ghizer. Each category is followed by subcategories, discussed further.

#### Views of stakeholders about suicide

Participants shared their different views on suicide in Ghizer.

*“Suicide obviously happens because of ill health; it’s not like it can’t be a reason but majorly all of this happens because of a lack of faith in God. This is the world, why we have been sent to this world, Allah SWT said in the Quran that we will test you through wealth, children, and earthly possessions. Means we will be tested through various means, now humans are sent here to live, not to take their lives” (P10).*

*“It is common in Ghizer due to the environment we have made; people are trying to copy and follow each other. Yea it has become a fashion or culture in Ghizer” (P01).*

*“It is a gender issue, and suicide is more common in females” (P02).*

*“It is said that if a person commits suicide he has a murder behind that act and that murder is named as society” (P04).*

*“In our area, after 1980s suicide cases were reported but the reporting rate of suicide cases in 1980s was less? But after 2000 and onwards the reporting rates of suicide cases were greater than before. Currently, 60% of cases are reported and 40% are under-reported” (P05).*

#### Perceived situations contributing to suicide

Participants highlighted situations leading to suicide in Ghizer, including high expectations from youth, low resilience ability, domestic violence, family dynamics and clashes between traditional and modern environments.

##### Competitive environment and high expectations from adolescents

Environment plays a vital role in an individual’s life. Analysis of data revealed that participants felt society was creating a very tough environment for children in their careers and they felt compelled to fulfill the wishes of parents by choosing the professions which they wanted. Stakeholders said:

*“Parents and people around them create an environment of competition. It is not possible for every child to win the competition, then parents send their child to that competitive environment and where so many other competitors are also present. Sometimes you cannot reach that level and feel like that you are useless and should have died” (P03).*

*“When parents visit their child’s school, they always ask about numbers and grades and they never ask about the behavior, friend circle and personal growth. Similarly, the teacher also discusses the academic performance with parents but not the behavior of the child” (P02).*

Majority of the participants expressed that parents expect more from adolescents in career achievement, and want that their children to follow the career path parents choose for them and for the children to excel in that discipline.

*“Parents want to make the child a doctor or engineer or commissioned officer but the child may not have the ability to do so. Allah may have blessed the child with other abilities but those abilities are suppressed by parents” (P05).*

##### Low resilience ability

Analysis of data showed that one of the causes of suicide was due to the lack of resilience in individuals in Ghizer. Most of the participants highlighted a lack of tolerance in people and they preferred to take a quick decision in every aspect of life including educational, professional or personal. A participant expressed his perceptions as follows:

*“There is a major issue of inferiority complex and frustration. Children and people are frustrated by the resources other people have like a smartphone, designer dresses, cars, makeup, etc.” (P07).*

In addition, religious leaders of different communities residing in Ghizer, expressed their views about lack of resilience ability in people as follows:

*“Suicide occurs due to lack of immunity and tolerance in an individual. Impatience is a basic reason for suicide. Impatience creates reasons for a person that led him towards this path. That’s why Allah SWT repeatedly said about patience; patience, and righteous path. If a person deviates from the righteous path and patience, then it creates evil like suicide” (P10).*

*“18-25 years old are at higher risk because these people are more emotional, not strong physically and emotionally” (P09).*

Some participants stated that if we allow frustration and negativity to control the mind and thinking it will ultimately affect our resilience power.

*“If everyone starts allowing these minor issues to dominate their mind and sense of thinking he or she will definitely get frustrated. It’s how you perceive things around you, like your relationship with your father, sister, brother, husband, wife, or mother, how you understand the expectations and needs of these relations. I believe that every person has this button in his heart, by pressing that button you can illuminate the surrounding things” (P07).*

##### Domestic violence (DV)

Domestic violence as a major cause of suicide emerged from the data analysis. Most of the stakeholders told that women were victims of violence (verbal or physical) from parents, siblings, husband, and in-laws (if married). Additionally, few participants shared their views about the effects of domestic violence on children.

*“We came to know that, mother-in-laws are doing violence and addicted husbands are doing physical violence on their wives” (P08).*

*“If the brother or father of a girl comes to know about her love affair, they started to do verbal and physical violence instead of finding the solution to the problem in a polite manner. In this way, the girl became hopeless and considers her life as useless in the world. She feels like a stranger and alone in the society, and thinks that suicide is the final solution to end her life from the problems” (P0*2).

One participant shared that they tried to find out the causes of suicide through a survey and came to know that violence, including domestic violence was one of the main causes. He elaborated:

*“Actually, we found the factual reason for suicide through investigation in our area is “cruelty”. We found that parents are not doing cruelty with their children. In two, three cases of suicide, we came to know that, mothers-in-law are doing violence. In six to seven cases, addicted husbands are doing physical violence on their wives” (P08).*

“*Women who are young or old are suppressed and tried to fulfill all the demands made on them. Husband do this, brother does the same, even the son also does the same with his mother and behaves badly, no matter what age- 50 years or 60 years” (P02).*

##### Family dynamics

Data analysis found that the family system has declined and the relationship among family members is weak. The behavior of elders towards youngsters in a family was either very rigid or consisted of fear, restricting them to share their problems and feelings.

*“The behavior of parents with their child or child-parent relationship might be declining. Here the behavior of parent with the child is either very strict or consist of fright, it is not a friendship…one of the biggest problem is that we don’t have good relationships among siblings, I don’t know about what my brother is doing or what my sister is doing. Parents don’t have an idea about where their son or daughter is going or what they are doing. They come to know when already the problem is out of control, so the biggest issue is that we have weak mutual coordination among family members or among people in society” (P03).*

*“We are living village type of areas and have a village like a family system where elders cannot understand the feelings of youngers which creates a gap between them, and this could be one of the reasons for committing suicide” (P04).*

Some participants discussed failure in love, relationship breakups, and relationship issues with the in-law’s family after marriage that they are also becoming the main reasons behind suicidal cases.

*“The reason for suicides in elderly is that they are doing the job of managing their family well till the age of 40 years of age. When their children grown up, there exist relationship issues between husband and wife, man (husband) tolerates this situation but it is difficult for the female to accept it. Husband and wife lived together for 20 to 40 years, they don’t need much support of each other at a young age but wife needs support at an old age from the husband. But due to family issues, she doesn’t receive the support from him. She becomes depressed and ultimately takes her life” (P10).*

*“There was a boy, he was in love and wanted to marry a girl, the girl also agreed but their parents were not willing, so he poisoned himself” (P07).*

#### Environmental factors

This category is divided into two sub-categories, which are discussed further one by one.

##### Clashes between traditional and modern environment

Almost all the stakeholders stated that Ghizer district was going through a transitional period which was good for the society, but while there were benefits of modernity it was also a source of risk for suicides in adolescents. They further added the reason as that people didn’t accept the modern transition completely but only partially which created conflicts for the younger generation. A stakeholder described as below:

*“Ghizer is going through a transition period and changes are coming over here but our role is missing. These changes are coming from other ways, such as via media or internet, that overwhelms the society and has even reached in our homes and our kids have become involved. We already have conflicts of culture, as well as tradition, arising here, and kids are stuck in between. They put one leg on modern society and another leg in traditional society and are unable to understand that “what to do and where to go” (P03).*

*“The cultures of country, city or northern area are challenging nowadays, you can say we are comparing the cultures and religion. Years ago, when Mukhi Sahib in Jamat Khana or Imam in Masjid or Sheikh gave advice about any topic people practiced it or if they didn’t practice they didn’t think about it. But now when people hear a lecture in Masjid or Jamat Khana, they first think about it, discuss with each other and search for it on social media which creates mental clashes. Due to this mental clash, they become mentally disturbed and when don’t find any results, commit suicide” (P05).*

*“When our sisters or children moved to the city, for study purpose or for the job and returned to the village, they could not adjust themselves to the environmental changes and became mentally disturbed” (P11).*

Some stakeholders also stressed that people who came from different cities to our area and conducted sessions on counseling have negative impacts on society due to cultural differences. One participants narrated

*“People from different NGOs visit and stay here for 7 to 10 days. They come in a high profile way, have a luxurious meal and leave. This increases suicide rates because people commit suicide after attending them, as to why they don’t have such facilities and status. It creates feelings of low self-esteem and related issues on their mind and people do commit suicide” (P05).*

##### Influences of media on society

The results showed that the media has both positive and negative effects on an individual’s life in modern society. Most of the stakeholders depicted the negative impact of media on a person. However, few participants portrayed the positive role of media in suicide prevention.

*“On social media platforms people post pictures and tell fake stories, then our young generation is getting inspired by them and they think that if anything goes wrong we always have this solution (suicide) in our hands. Children observe everything very minutely; they make their perceptions according to their surroundings” (P07).*

*“There is gap created between the child and the parents and it has many reasons, technology is playing its role in this, parents are either busy with their work or then technology innovated activities, social media, Facebook, YouTube, etc., and the gap is affecting the child grooming” (P06).*

*“Media is very active in reporting suicide cases in Ghizer which has both positive and negative impacts- it is a good thing and based on the reporting, people or NGOs will work on it…Media reporting will help to plan interventions based on the reporting and reporting will give signals to NGOs, researchers and other accountable persons in society to do interventions” (P03).*

A participant highlighted hurdles for reporters in the area:

*“Reporting is being done to reveal the hidden facts, on behalf of our society. Sometimes people only show hate, like on social media platforms, which means that we also have resistance among people that why they only have to talk about negative aspects.” (P07).*

### Theme 2: role of the health system in suicide prevention

This theme is subdivided into two categories: major hurdles in the health system and lack of MH services in the available health system. Category one has sub-categories that are discussed further in the following paragraphs.

#### Major hurdles in the health system

Most stakeholders stated that there were barriers in the health system for suicide prevention, like lack of awareness about MH issues and unpredictability of suicidal thoughts.

##### Lack of awareness about MH issues

Most participants emphasized a lack of awareness about MH issues in the area. They further elaborated that people did not consider MH issues as diseases:

*“I think in our area 60% of the people will consider mental issues as mental illness and 40% will consider influences of spirits on the individual. So a mentally ill patient is considered being possessed by spirits” (P04).*

*“We do not have a proper mechanism of making people aware of this. Other issues are, people resist telling such problems or disclose any disorder or disease which they have, as they feel this might increase social pressure on them and they would not be accepted because it is too dangerous the society” (P06).*

Participants further elaborated the lack of health education in primary health system:

*“There is a lack of education in the health system. There is also a lack of professionals dealing with psychiatric illness, unavailability of doctors, and lack of awareness about psychiatric issues” (P02).*

*“According to my knowledge, there is a lack of information about MH issues. If we take the patient who has MH issues to a nearby hospital, the doctor will say, try to keep the patient happy. If the daughter says that I want to marry a certain person, we have to agree and let her marry, because there is no other option for making her happy” (P09).*

Some participants reported that suicides cases are more in Ghizer because people give more preference to secular than religious education. They added people are trying to follow each other and consider suicide as an easy way out of their problems. For e.g. two stakeholders said:

*“It is due to lack of awareness and religious education. Parents are not guiding their children, and it is not discouraged in schools” (P01).*

##### Unpredictable suicide behavior

Almost all the stakeholders stated that it was difficult to predict suicide because they had come across such cases where individuals were absolutely fine a day before the suicide. This was a great challenge in the prevention of suicide in Ghizer. Stakeholders stated:

*“One of my cousins who committed suicide in the near past, was well and ok a few days before I met him. Suddenly I was informed that he has committed suicide. So it’s really difficult to identify such people.” (P06).*

*“We cannot stop the person who wants to and is planning to do suicide. We come across such cases where the individual was perfectly fine and behaving normally before committing suicide” (P05).*

*“In our society people don’t know how a person is going to commit suicide; they are unable to recognize the individuals. The person who has to commit suicide finds a hidden place, closed room or goes to a desolate place and ends his life” (P04).*

However, few participants felt that the person who was planning suicide may visit the nearest health center but with other complains. For e.g. one participants said:

*“A girl who visits the local doctor will never tell that she is going to commit suicide or that a boy cheated on her. Instead she visits with the complaint of a headache” (P01).*

#### Lack of MH services in the available health system

Analysis of the data showed all participants reported the lack of MH facilities in the area. Existing health services, either public or private were not efficient in suicide prevention due to a shortage of resources. Participants said:

*“I don’t think so; existing health services are effective at no more than 5%. They have failed in terms of suicide control, the rate was decreasing but now the rate touches the sky and at least 10 to 20 mothers or sisters or children are committing suicide per year” (P10).*

*“We don’t have any effective existing MH facilities that can be helpful in suicide prevention, because if we could have facilities rates of suicide would decrease but suicide rates are increasing day by day in Ghizer” (P11).*

*“How could I explain to you about the public hospitals, the working staff don’t even take accurate blood pressure readings so how could they can they assess mental status… How can a doctor who is assessing 100 to 150 patients, assess the mental level of patients”? (P05).*

Majority of stakeholders described the lack of infrastructure and resources including human resources and general health resources for MH services in the area.

*“My cousin is not mentally well; we used to take him to Abbottabad for his treatment due to unavailability of MH facilities here” (P08).*

*“We don’t have any MH hospital or clinic in whole Gilgit-Baltistan. People used to take their loved one to Dudial (Mansehra), which is 354 kms on Karakorum highway, if they have any MH problems” (P09).*

*“We don’t have any center for psychological or mental issues in Ghizer.” (P03).*

### Theme 3: challenges for the health system in suicide prevention

Data analysis revealed that there are major challenges faced by the health system of district Ghizer.

#### Lack of collaboration across sectors

Almost all stakeholders reported that different institutions are working on suicide prevention in Ghizer which is a positive step but these organizations are working in isolation, without coordination, leading to ineffectiveness of suicide prevention programs.

*“Neither any institution has bothered to take any action or investigate, nor taken any action yet. We have a social welfare organization and it is their duty to identify social issues and provide guidance to the public. I didn’t find the contribution of the private and public organization in a single case till now” (P05).*

*“We left all things out of control; every aspect of society is weakened. When we find out about the incidents we go there and do mourning but don’t do anything before the incident which we should do” (P03).*

In addition, some participants voiced that there should be written guidelines/directions for suicide prevention. Participants said that a committee was formed by Chief Minister of Gilgit-Baltistan for suicide prevention and directions were given, including, a policy for medical autopsy of each suicide case, which should be implemented in the district.

*“Mutual collaboration is lacking among the organizations working in different areas of Ghizer... clear-cut policy and instruction were given to do an autopsy of every suicide case” (P05).*

*“Till now we didn’t see any institution or any NGO which is working in suicide prevention. If we have a MH strategy, then we could have control over on suicide rates which are alarmingly increasing day by day in Ghizer” (P11).*

*“We all know that this problem is a part of our society and it exists on every level. Until or unless the whole society is not willing to eradicate this issue we can’t resolve it. What we do is we make committees and organize a session and that’s it” (P03).*

*“Social officer is designated to look at social issues and part of the Ministry of Special Education. So the Ministry should give the task to the officer” (P01).*

#### Un-availability of MH professionals

In data analysis, most participants reflected that there is unavailability of MH experts including psychiatrists, psychologists, counselors and MH nurses in the district.

*“We do not have psychiatrists, there is a lack of counseling and we do not have a mental hospital. We do not have a single psychiatrist in the whole GB” (P01)* … “*We have doctors in our area who are just MBBS and dealing with other health issues. We don’t have MH specialists therefore we have to travel to other cities in Pakistan” (P08).*

*“According to my experience, we don’t have any center for psychological or mental issues in Ghizer. Neither have an expert from whom we can get help nor have such guidelines in schools or colleges” (P03) … “We don’t have any MHcare professionals who can help people suffered from tension or depression” (P09).*

Importance of research related to MH issues in the area was also highlighted:

*“Health system doesn’t have a researcher and MH specialists to handle mental issues in the area, this is a big challenge” (P10).*

#### Financial issues

Most of the stakeholders highlighted financial needs for the health system in terms of MH issues and suicide prevention. They reported that financial constraints are very crucial and require the attention of the health department of Gilgit-Baltistan.

*“There is no budget for MH issues in the health department and, no one can solve this issue free of cost” (P01) … “Even the experts are not ready to give services until they receive a good (handsome) money. A huge amount of money is always required to initiate even a small program” (P02).*

Some participants stressed on budget allocation for suicide prevention and that no work can be done without financial resources.

*“I think we don’t have the budget for suicide prevention in existing resources/facilities. It is a very serious issue, there should be a budget allocation and specific budget should be allocated for the prevention of this issue” (P04) … “I would like to tell that people in our society either illiterate or educated, will not be ready to do any work without a huge amount of money”*

## Discussion

This research study explored the perceptions of stakeholders about the role of the health system in district Ghizer in Gilgit-Baltistan, northern Pakistan. It explored their perspectives about suicide as a social issue, the role of the health system in suicide prevention and the major challenges for the health system in suicide prevention. The following section discusses the results in the context of the emerging themes of the research, supported by relevant literature.

### Suicide as a social issue

To understand the role of the health system in suicide prevention, it is important to explore the perceptions of stakeholders, as important influencers in prevention programs. This study highlighted a number of social issues in Ghizer such as the high expectations of parents in educational, social and marital matters that created mental pressure and made them vulnerable to suicidal behavior. Other studies show similar findings [27]. The family acts as a buffer against stressors in an individual’s life and a healthy positive family relationship is protective from depressive feelings and suicidal thoughts [19]. In recent years the child-parent relationship appears to have deteriorated in the family system in Ghizer. Our study showed that young people in Ghizer may lack resilience making them vulnerable to depression and suicidal behaviors [28].

Domestic violence leaves the victim with feelings of helplessness, decreased self-esteem, hopelessness, lack of trust, feelings of oppression, making them more susceptible to suicide [29]. There are reports that prevalence of DV may be high in GB [13] and this may be reflected in high rates of suicides in women [30]. Similarly, studies also show that childhood experience of violence (sexual, physical, or emotional) or being exposed to violence in the home has a substantial effect on their health. Childhood experiences to violence is a risk factor for a variety of risky behaviors including smoking, drug abuse as well as suicidal behaviors [21, 31].

Environmental factors such as social transition and effects of media have a profound effect on vulnerable individuals and this is reflected in the views of our stakeholders in the context of Ghizer. Other studies have shown similar results, particularly the role of media in the way suicide incidents were reported [28, 32].

### Role of the health system in suicide prevention

Early identification and treatment of many mental disorders, especially depression can prevent an individual from progressing on the suicidal pathway [33]. Our study found that the lack of awareness about MH issues is the major hurdle in the prevention of suicide in Ghizer.

There are consistent findings that majority of suicides take place in LMICs, where there is also high prevalence of mental disorders but also, paradoxically there is lack of resources to address these and many mental disorders remain untreated [22, 34]. Participants in our study highlighted the poor health and MH facilities and shortage of resources.

There extreme shortage of MH workforce and people were not receiving appropriate care as a result [35]. There is need for training of healthcare as well as non-health personals (lay workers, caregivers, affected individuals) in detection and referral of people with MH problems [36]. The system of investigation and diagnosis of suicide cases in Pakistan needs improvement urgently through of police, medical doctors, forensic medical, and medico-legal personnel [37].

### Challenges for health system

Suicide is a highly challenging problem and prevention requires effective collaboration of all organizations and (public or private) and different stakeholders [33]. Our study showed that in Ghizer, organizations were working in isolation and lacked coordination. Our study also revealed that although a forensic autopsy was carried out, a psychological autopsy to determine the underlying psychological causes was not there [38–40]. .

Unavailability of MH professionals in Ghizer is one of the biggest challenges for the health system and people had to travel long distances to another city to receive mental healthcare. In suicide prevention majority of persons dying by suicide have been in contact with health services in the weeks before the suicide [22, 36].

Lack of financial resources was another major challenge in the prevention of suicide in Ghizer. This is reflected in only 0.4% of healthcare budget being allocated to MH. There are no social insurance organizations for mental health problems in Pakistan [22]**.**

### Strengths of the study

To the best of the researcher’s knowledge, the study is first to be done in the context of Ghizer, Gilgit-Baltistan on perceptions of stakeholders in suicide prevention regarding the role of the health system in Ghizer, GB, northern Pakistan. In addition, a diverse group of stakeholders was selected, this comprised variation who varied in their age, profession, education level and experiences. Data was collected by the researcher herself and the direct involvement during the research process uplifted ensured consistency of findings the quality of research.

### Limitation of the study

The study was only limited to Ghizer district due to constraint of time and the district has its own unique geography, population and health system. Hence results cannot be generalized to other districts in GB or rest of the country. However, it can be conducted in other districts, for a deep understanding of the phenomenon because suicides cases have also been reported from all districts of GB. Majority of the participants were males in a higher position, which may have skewed the findings. Restricted researchers thorough inquiry. Moreover, one of the major limitation was less number of participants, we conducted 12 interview from 12 stakeholders there may be less saturated information as the group was diversified in nature.

### Recommendations

The government should prioritize MH and suicide prevention. Create awareness about MH in the community through electronic and print media as well as community awareness sessions. In addition, introduce school MH programs for early identification of risk factors among adolescents and provide facilities for youth that can help in strengthening their resilience and coping power. Also, a program for healthy parenting is suggested.

### Policy implications

Findings of the study can inform policy for a comprehensive multi-sectoral suicide prevention strategy involving educational, police, health and social welfare department, as well as civil society, NGOs, and religious leaders. Furthermore, improving system of registering and diagnosing suicide, including forensic as well as psychological autopsy in every suspected case of death by suicide.

### Future research

More ethnographic studies to explore the inter-sectoral collaboration and nature of work on suicide prevention in the district Ghizer. Replication of this study can be done in other areas of GB as well as other areas of the country.

## Conclusion

To the best of the researcher’s knowledge, the study is first to be done in the context of Ghizer, Gilgit-Baltistan on perceptions of stakeholders regarding the role of the health system in suicide prevention. The findings revealed that the role of the health system is lacking in suicide prevention. There are multiple challenges for the health system including a lack of awareness on mental issues, shortage of human/financial resources and lack of collaboration in the community. There is a need for reviewing existing policies and strategies to overcome the existing challenges for the effective prevention of suicide in the area. This study the need for creating awareness about MH issues, introduction of school health programs, parent counseling and strengthening of the health system by allocating the suitable resources for MH issues and suicide prevention.

## Supplementary information

**Additional file 1.**

**Additional file 2.**

## Data Availability

The datasets generated and analyzed during the current study are not publicly available due to participant confidentiality but are available from the corresponding author on reasonable request.

## Abbreviations

AKHS, P: Aga Khan Health Services Pakistan
LMICs: Low and middle-income countries
PTSD: Post-traumatic stress disorder
AKHB: Aga Khan Health Board
WHO: World Health Organization
NGOs: Non-governmental organizations
TBI: Traumatic brain injury
DHO: District Health Officer
ERC: Ethical review committee
DC: Deputy Commissioner
GB: Gilgit-Baltistan
MH: MH
SWT: Arabic: Subhanahu wa ta’ala (English: The most glorified, the highest)

## References

[1] World Health Organization (2012). Public health action for the prevention of suicide: a framework.

[2] World Health Ogranization (2019). Suicide- key facts.

[3] Gearing RE, Lizardi D (2009). Religion and suicide. J Relig Health.

[4] Pakistan Bureau of Statistics (2017). 6th polpulation and housing census: government of Pakistan.

[5] Mirza I, Jenkins R (2004). Risk factors, prevalence, and treatment of anxiety and depressive disorders in Pakistan: systematic review. Bmj..

[6] Khan MM (2016). Economic burden of mental illnesses in Pakistan. J Ment Health Policy Econ.

[7] Khan MM, Reza H (2000). The pattern of suicide in Pakistan. Crisis Tor.

[8] Khan MM, Naqvi H, Thaver D, Prince M (2008). Epidemiology of suicide in Pakistan: determining rates in six cities. Arch Suicide Resh.

[9] Abdullah M, Khalily MT, Ahmad I, Hallahan B (2018). Psychological autopsy review on mental health crises and suicide among youth in P akistan. Asia Pac Psychiatry.

[10] Ghizer Times (2014). Number of suicide in Ghizer District of Gilgit Baltistan from 2007 to 2012.

[11] Rahnuma B, Fangtong J, Khan MA, Saddique K, Ahmad I (2017). Causes of suicide in Gilgit-Baltistan region. Eur Acad Res.

[12] Sher S, Dinar H (2015). Ethnography of suicide: a tale of female suicides in district Ghizer, Gilgit-Baltistan. Explor Islamabad J Soc Sci.

[13] Department of International Development (2000). Assessmet of root causes of suicide among women in Ghizer, district of nothern areas of Pakistan.

[14] Dodani S, Zuberi RW (2000). Center-based prevalence of anxiety and depression in women of the northern areas of Pakistan. J Pak Med Assoc.

[15] Sugimoto-Matsuda J, Rehuher D (2014). Suicide prevention in diverse populations: a systems and readiness approach for emergency settings. Psychiatr Times.

[16] Naz S, Memon A, Haq M, Nadeem U, Jamal G, Khan A (2016). Pakistan district education ranking Islamabad: Alif Ailaan.

[17] Speziale HS, Streubert HJ, Carpenter DR (2011). Qualitative research in nursing: advancing the humanistic imperative: Lippincott Williams & Wilkins.

[18] Keugoung B, Kongnyu ET, Meli J, Criel B (2013). Profile of suicide in rural Cameroon: are health systems doing enough?. Tropical Med Int Health.

[19] Samm A, Tooding L-M, Sisask M, Kõlves K, Aasvee K, Värnik A (2010). Suicidal thoughts and depressive feelings amongst Estonian schoolchildren: effect of family relationship and family structure. Eur Child Adolesc Psychiatry.

[20] Bustamante F, Urquidi C, Florenzano R, Barrueto C, Hoyos J, Ampuero K (2018). El programa RADAR para la prevención del suicidio en adolescentes de la región de Aysén, Chile: resultados preliminares. Rev Chil Pediatr.

[21] Anda RF, Felitti VJ, Bremner JD, Walker JD, Whitfield C, Perry BD (2006). The enduring effects of abuse and related adverse experiences in childhood. Eur Arch Psychiatry Clin Neurosci.

[22] World Health Ogranization (2009). WHO-AIMS report on mental health system in Pakistan.

[23] Asif AF (2017). Healthcare challenges in Gilgit-Baltistan: the way forward. Pak J Public Health.

[24] Polit DF, Beck CT (2008). Nursing research: generating and assessing evidence for nursing practice.

[25] Creswell J (2013). Qualitative inquiry and research: choosing among five approaches.

[26] Polit DFB, C.T (2008). Generating and assessing evidence for nursing practice.

[27] Nolle AP, Gulbas L, Kuhlberg JA, Zayas LH (2012). Sacrifice for the sake of the family: expressions of familism by Latina teens in the context of suicide. Am J Orthop.

[28] Kamble R (2015). Resilience, suicidal ideation, depression and adolescents. Int J Educ Psychol Res.

[29] Kavak F, Aktürk Ü, Özdemir A, Gültekin A (2018). The relationship between domestic violence against women and suicide risk. Arch Psychiatr Nurs.

[30] Khan MM, Ahmed A, Khan SR (2009). Female suicide rates in Ghizer, Pakistan. Suicide Life Threat Behav.

[31] Mercy JA, Krug EG, Dahlberg LL, Zwi AB (2003). Violence and health: the United States in a global perspective. Am J Public Health.

[32] Kwok SY (2014). The moderating role of emotional competence in suicidal ideation among Chinese university students. J Adv Nurs.

[33] Dorji G, Choki S, Jamphel K, Wangdi Y, Chogyel T, Dorji C (2017). Policy and governance to address depression and suicide in Bhutan: the national suicide-prevention strategy. WHO South East Asia J Public Health.

[34] Bruckner TA, Scheffler RM, Shen G, Yoon J, Chisholm D, Morris J (2011). The mental health workforce gap in low-and middle-income countries: a needs-based approach. Bull World Health Organ.

[35] Bono V, Amendola CL (2015). Primary care assessment of patients at risk for suicide. J Am Acad PAs.

[36] Scheffler RM, World Health Organization (2011). Human resources for mental health: workforce shortages in low-and middle-income countries.

[37] Shekhani SS, Perveen S, Akbar K, Bachani S, Khan MM (2018). Suicide and deliberate self-harm in Pakistan: a scoping review. BMC Psychiatry.

[38] Zhang J, Xiao S, Zhou L (2010). Mental disorders and suicide among young rural Chinese: a case-control psychological autopsy study. Am J Psychiatr.

[39] Khan MM, Mahmud S, Karim MS, Zaman M, Prince M (2008). Case–control study of suicide in Karachi, Pakistan. Br J Psychiatry.

[40] Milner A, Sveticic J, De Leo D (2013). Suicide in the absence of mental disorder? A review of psychological autopsy studies across countries. Int J Soc Psychiatry.

