# Supplementary material for: Perceptions of stakeholders about the role of health system in suicide prevention in Ghizer, Gilgit-Baltistan, Pakistan
Source: BMC Public Health. 2020 Jun 23;20:991. doi: 10.1186/s12889-020-09081-x (PMC7313136; doi:10.1186/s12889-020-09081-x)
Supplement: Supplementary file 1 — Additional file 1. [file 12889_2020_9081_MOESM1_ESM.docx]

**Additional File A Interview Guide (English)**

| *Title: Perception of Stakeholders about the role of Health System in Suicide Prevention in Ghizer, Gilgit-Baltistan.* | |
| --- | --- |
| **Category No.01: Demographic Information** | |
| Name________________ Age_________________  Gender ______________ Qualification_______________  Name of Institution__________________ Position____________________  Experience________________ Type of Institution_____________  E-mail address_________________________ Contact Number____________________  Date of Interview____________________ Time of Interview___________________ | |
| **Category N0.02: Perceptions about Suicide** | |
| 1 | What are the major health issues in Ghizer?  **Probes:** what are the mental health issues? |
| 2 | Could you please share how you see suicide in Ghizer?  **Probes:** Is it a health issue? Social issue or Personal issue |
| 3 | What do you think, which are the most vulnerable groups for suicide? and why?  **Probes:** youth, married women, men or any other? |
| 4 | What are the main factors influencing suicidal behaviors in Ghizer?  **Probes:** Cultural beliefs and attitudes, Family issues, Gender and age issues, Mental health, marital issues (love marriage/arranged marriage or second marriage of husband), other issues.  *(Note: additional probing question for healthcare professionals)*  What type of causes you deal commonly in your experience? |
| **Category No.03: Available Mental Health Services** | |
| 5 | In your knowledge, what are the existing mental health strategies/services available in the area?  **Probes:**  Awareness (efforts have been made to address the stigma)  support system for the bereaved families  Treatment of diagnosed mental issues  Number of mental healthcare professionals (doctors, nurses or midwives)  Available mental health centers, where people can go and avail the mental services |
| 6 | Could you please elaborate the accessibility of mental health services to people?  **Probes:** within reach/ too far, Is the fee affordable for services? |
| 7 | How well are the current mental health services doing in terms of preventing suicide?  **Probes:**  Identification of symptoms before the event of suicide  Signs of Depression, stress and anxiety  *(additional probing questions for healthcare professionals only)*  What are the mental scales used to identify risk individuals? |
| 8 | Once the symptoms are identified, what actions do you take?  **Probes:** Support in treatment, Counseling sessions  Is there any program for parent Counseling (identified youth)?  *(additional probing questions for healthcare professionals only)*  How frequently follow ups and counselling sessions are conducted?  How do you track identified individuals for follow up visits, if they don’t visit? |
| 9 | What do you think, what are the difficulties to tackle the identified at risk cases?  **Probes:** unavailability of Psychiatrists, Quality of time spend by psychiatrists, unavailability of medicine, referrals system for identified cases, Community Preferences on religious treatment |
| 10 | In your opinion, what is the lacking in available mental health services?  **Probes:** Trained mental Healthcare professionals, lack of awareness in community to avail services, available mental services are expensive |
| **Category No.04: Challenges and Coordination** | |
| 11 | What are the major challenges facing your community to prevent suicide cases? |
| 12 | What are the challenges faced by health system to Tackle the Issue of Suicide?  **Probes:** Lack of Strategy, Commitment of leadership, Lack of Infrastructure, financial issues |
| 13 | In your opinion, what are barriers that may be affecting the use of local mental health services?  **Probes:** Stigma, Lack of awareness among community about mental illness, Services are not available/non affordable, More belief in traditional faith healers |
| 14 | What do you think, what would help to remove barriers that may be affecting the use of local mental health services by the community as a whole? |
| 15 | How you will explain the collaboration in the community among various organizations to prevent suicide in the area?  **Probes:**  Among Hospitals public and private  Between Clinics, hospitals and Pharmacies  Between Law enforcement agencies, hospitals and social organization, Among Schools, primary health centers, Other local health providers, for example homeopaths  **(Note:** *Question No.15 and 16 are not applicable for parents and youth***)** |
| 16 | In your opinion, what is the role of your organization to deal with the issue? |
| **Category No.05: Suggestions** | |
| 17 | Would you like to suggest anything to prevent suicide cases? |
| 18 | How the available resources can be utilized to strengthening the system to prevent suicide? |
