# Supplementary material for: Perceptions of stakeholders about the role of health system in suicide prevention in Ghizer, Gilgit-Baltistan, Pakistan
Source: BMC Public Health. 2020 Jun 23;20:991. doi: 10.1186/s12889-020-09081-x (PMC7313136; doi:10.1186/s12889-020-09081-x)
Supplement: Supplementary file 2 — Additional file 2. [file 12889_2020_9081_MOESM2_ESM.docx]

**Research Design:**

The qualitative descriptive design provides an opportunity to explore and describe in-depth the understanding of a concept or phenomenon and assists in unfolding human experiences (1). The selected design was found to be ideal for this study as this design enables the researchers to understand the needs of a specific population or to achieve the desired outcome in order to develop appropriate interventions to benefit the population (2). “Exploratory descriptive qualitative researchers identify a specific lack of knowledge that can be addressed only through seeking the viewpoints of the people most affected” (2).

As various stakeholders come under the health system, there will be the diversity of perceptions and understanding about the capacity and role to deal with the emerging trend of suicide. Therefore, it will help me to describe the phenomena and barriers/gaps in detail and will expand my understanding. In this study, no any prior framework for data was used except the literature review to build concepts on the research process. Moreover, literature also supports that not every qualitative descriptive exploratory design need a prior conceptual framework, the literature review of a study often labeled as a conceptual framework.

**Establishing Rigor of the Study:**

The criterion of Lincoln and Guba includes credibility, confirmability, dependability, and transferability.

**Confirmability:** The identification of limitations and the researcher’s beliefs and assumptions will enhance conformability. It is concerned with establishing that the data specify the information provided by the respondent and interpretations of data are not manipulation of the researcher’s thinking (3). To make sure conformability reflective logs were written to condense the biases in the interpretation and to isolate the feelings of the investigator. Furthermore, the analysis was done under the supervision of supervisor and committee members.

**Credibility:** The term credibility refers to the significance and trustworthiness of the research findings (3), to ensure the credibility of this study, an interview guide (Appendix E) was formulated in consultation with the supervisor and committee members. There was the addition of Intended and unintended probes in the interview guide, after piloting it with two participants. Checks with the respondents were done when elucidation is needed. Non-verbal communications, as well as gestures, were recorded in the field notes to cater the reactions of participants during the recording of the interview. Further, triangulation of data would be accomplished by asking the same questions to different study participants.

**Dependability:** The term dependability is related with the reliability of study results. The dense description of procedure about the unique situation of the problem, triangulation and cod record procedure indicates the dependability of any study (4, 5).

**Transferability:** According to literature (5), “Transferability refers to the probability that study findings have meanings to other in a similar situation”. The transferability of the study can be accomplished through a dense description of the data that enables the application of study in similar settings (4). The finding of this study might be transferable to the other settings where contexts are similar. For example, other districts of Ghizer, Chitral, Swat and Quetta Pakistan. In addition to that, the study findings will be disseminated through publications and poster presentations at the national and international level. Moreover, summary of the study results will be send to the participants of the study and the hard copy of this thesis will also be kept in the AKU library.

**References:**

1. Wood M, Ross-Kerr J. Focused Ethnography, Basic Steps in Planning Nursing Research From Question to Proposal. Jones & Bartlelt Publishers, Canada; 2011.

2. Grove SK, Burns N, Gray J. The practice of nursing research: Appraisal, synthesis, and generation of evidence: Elsevier Health Sciences; 2012.

3. Polit DF, Beck CT. Nursing research: Generating and assessing evidence for nursing practice. 2008.

4. Krefting L. Rigor in qualitative research: The assessment of trustworthiness. American journal of occupational therapy. 1991;45(3):214-22.

5. Speziale HS, Streubert HJ, Carpenter DR. Qualitative research in nursing: Advancing the humanistic imperative: Lippincott Williams & Wilkins; 2011.
